# Lignocellulosic Biomass-Based Metal–Organic Frameworks: A Sustainable Frontier for Advanced Wastewater Remediation

**DOI:** 10.3390/polym18101235

**Published:** 2026-05-19

**Authors:** Aparna Sudarsana Babu, Florian Zikeli, Debora Puglia

**Affiliations:** Civil and Environmental Engineering Department, UdR INSTM, University of Perugia, 05100 Terni, Italy; aparna.sudarsanababu@dottorandi.unipg.it (A.S.B.); debora.puglia@unipg.it (D.P.)

**Keywords:** lignocellulosic biomass, metal–organic frameworks, composites, dye-removal, wastewater renovation

## Abstract

The emerging demand for water pollution control has driven a significant interest in advanced porous materials for sustainable and effective wastewater treatment technologies. Metal–organic frameworks (MOFs) have been employed as promising substrates due to their versatile properties, especially their high surface area, tunable properties, and chemical functionality. However, their practical applications are often limited by poor aqueous stability, instability during recovery, and high production costs. Lignocellulosic biomass (LCB) is an abundant, low-cost, and renewable resource, primarily composed of cellulose, hemicellulose, and lignin, offering a sustainable solution for these challenges. This review critically examines the recent advances in design and applications of LCB-MOF materials for wastewater remediation. Several synthesis strategies, including *in situ* growth, ex situ impregnation, and post-synthetic modification, are systematically discussed in relation to their significance in enhancing stability, recyclability, and dispersibility of MOFs. The key, structural, morphological, and physicochemical properties of these LCB-MOFs were analyzed, along with their performance in removing organic dyes and heavy metal ions. Current drawbacks in long-term stability, scalability, and real-world wastewater performance are highlighted. Overall, LCB-MOFs demonstrate a promising class of sustainable materials that align with the principles of the circular economy and green chemistry, making them ideal for next-generation wastewater remediation technologies.

## 1. Introduction

### 1.1. The Global Challenge of Wastewater Pollution

Access to safe drinking water is a vital human right that has become compromised in many places in our industrial world. Water pollution is now a global issue, threatening the environment and life on Earth, and occurs from various sources, including agricultural, domestic, and industrial activities, which release harmful toxic effluents, like heavy metal ions, dyes, and pharmaceuticals [1]. Even if the concentration of these pollutants was very low, untreated water could lead to life-threatening consequences for the aquatic system and various health problems, including carcinogenic impacts on human life [2,3]. Several treatment strategies have been developed to address water pollution in order to limit the hazardous environmental impacts of pollutants, such as coagulation or ion exchange, and adsorption [4]. Among them, adsorption processes proved to be the most efficient removal techniques, with the highest removal rates [5,6,7]. Adsorption processes enable the straightforward removal of heavy metal ions and toxic dyes from wastewater using different mechanisms, such as electrostatic interactions [8], H-bonding [9], and π–π interactions [10]. High surface area and porosity, economic stability, and reusability are the desired characteristics of an efficient and environmentally friendly adsorption material.

A wide range of conventional adsorbents, such as activated carbon, zeolites, metal oxides, and bio-based materials, has been widely used for wastewater remediation due to their relatively low cost and ease of application [11,12]. Among these, activated carbon has demonstrated its immense potential in water treatment, but certain limitations, such as high regeneration cost, low selectivity for inorganic ions, and limited adsorption capacity, hinder its utilization as a conventional adsorbent [13,14]. Similarly, zeolites suffer from a narrow pore size distribution, while metal oxides have low surface area, which limits their adsorption performance for various emerging pollutants [11]. In addition, conventional adsorbent materials suffer from disposal or regeneration problems, which can lead to secondary pollution [15].

Recently, biomass-based adsorption (or biosorption) processes have gained widespread attention due to their regenerative nature and high adsorption capacity [16]. Jain et al. successfully developed a biosorption method for removing methylene blue (MB) from textile wastewater using sugarcane bagasse, peanut hull, and orange peel, and their thermodynamic data showed that the adsorption was spontaneous and endothermic [17]. Research on carbon-derived compounds and other organic bio-adsorbents for water remediation also led to the emergence of novel nanoscale adsorbents that use nanoparticles as their major component, further improving surface area and porosity and facilitating heavy metal ions removal from water bodies [18,19,20].

Metal–organic frameworks (MOFs) are another class of organic compounds utilized for adsorption methods to remove pollutants from industrial wastewater [21]. MOFs are made by combining metal ions and organic ligands and are widely used due to their large surface area and porous nature, which provide high absorption efficiency and effective pollutant removal [22,23]. Recently, lignin derivatives such as ferulic acid, vanillin, or caffeic acid have been used for the synthesis of biobased MOFs to reduce the harmful effects and challenges, such as biocompatibility constraints associated with conventional organic ligands [24].

In this review, comprehensive information is collected regarding the combination of lignocellulosic biomass (LCB) and MOFs for wastewater remediation applications via adsorption or reduction processes for dyes or heavy metals. The constructive synergy between LCB and MOFs, particularly in wastewater treatment applications, is systematically explored. We focused on strategies for fabricating LCB-MOFs composites (*in situ* growth, ex situ deposition, etc.), structure–property relationships emerging from the LCB-MOFs interface, performance in water remediation applications targeting heavy metals and organic dyes, and practical challenges (stability, regeneration, scalability). Moreover, future directions of biobased and nanoscale MOF-type adsorbents are addressed.

### 1.2. Lignocellulosic Biomass: A Sustainable and Abundant Resource

**Figure 1:**
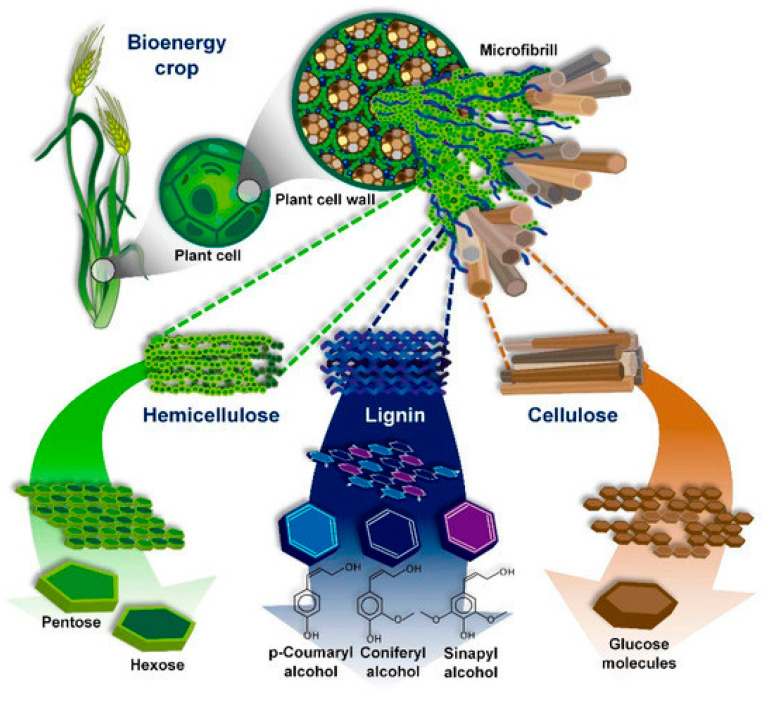
Schematic illustration of the arrangement of cellulose, hemicellulose, and lignin inside the cell walls of lignocellulosic biomass. Figure reproduced from Hernandez et al. [30], licensed under CC BY 4.0.

LCB is the most abundant renewable material on Earth and has the potential to serve as eco-friendly alternatives to fossil-based products [25]. LCB is available in the form of agricultural residues (e.g., rice and wheat straw, corn stover, sugarcane bagasse), energy crops (e.g., switchgrass, miscanthus), forest residues (e.g., leaf litter, fallen trees, trimmings), and industrial by-products (e.g., sawdust, paper mill waste) [26]. LCB consists primarily of three polymers, cellulose, hemicellulose, and lignin (Figure 1), which constitute around 85–90% of the biomass dry weight, providing structural integrity to plants [27]. Depending on the biomass source, species, plant age, and environmental conditions, the relative proportions of these three polymers vary, with cellulose accounting for 35–50%, hemicellulose for 20–40%, and lignin for 10–25% [28,29].

Cellulose is the primary component of biomass and consists of a linear polysaccharide built up by β-1,4-linked glucose units embedded in a matrix of hemicellulose and lignin. Cellulose contains highly crystalline regions that impart mechanical strength and require specific pretreatment conditions for their isolation via selective removal of amorphous regions [31,32,33]. Isolated cellulose is a preferred substrate for the conversion into nanocellulose, which can be particularly useful for MOF immobilization due to its high surface area and abundant hydroxyl groups [34,35,36].

Hemicellulose consists of branched heteropolysaccharides that provide an amorphous nature and flexibility. Hemicellulose-rich fractions are abundant in hydroxyl and carboxyl groups, making them valuable for use as functional scaffolds, structure-directing agents, or chemical precursors in fabricating composites [37].

Lignin is the third key component of lignocellulosic biomass, with its aromatic and hydrophobic nature, forming a rigid matrix that binds polysaccharide fibers together [38,39].

LCB has an inherent porous structure and an abundance of reactive surface functional groups, mainly hydroxyl and carboxyl groups, making LCB an attractive precursor for the synthesis of functional materials [40]. Due to the presence of these reactive moieties on the surface, LCB allows various chemical modifications that can improve compatibility with polymer matrices, enhance adsorption capacity, and fine-tune surface interactions, making it a promising platform for producing functional and high-performance nanoscale biopolymers [28].

### 1.3. Metal–Organic Frameworks (MOFs): A Revolution in Adsorbent Materials

MOFs are a class of crystalline porous materials formed by the coordination of metal ions, or metal-oxide clusters, with multidentate organic linkers to form a well-ordered network. They are renowned for their exceptionally large surface area and tunable properties, which can be modified by the presence of chemical functionalities on the ligands [41,42]. Compared to conventional porous absorbents such as activated carbon and zeolites, MOFs offer structural tunability of the pore environment, thereby increasing affinity for specific contaminants. Further, no sludge from wastewater treatment is produced as MOFs can be regenerated, maintaining their structural properties [11]. Recent studies have demonstrated that the critical properties of MOFs, such as pore size and connectivity, can be enhanced by carefully selecting metal ions and linkers. MOFs also support post-synthetic modifications, which enable the introduction of additional functional groups or ligand exchange without compromising the crystalline framework [41,43,44]. MOFs show great potential for different applications, such as sensing [45], supercapacitors [46], material separation [47], heterogeneous catalysis [48], targeted drug delivery [49], heavy metal ion removal [50,51], and adsorption [52].

The chemical features of MOFs make them exceptionally well-suited for wastewater treatment. Firstly, the surface area and porosity of MOFs provide a high adsorption capacity for a wide range of contaminants, including dyes, heavy metals, and organic pollutants [41]. Secondly, the chemical versatility of MOFs allows for different combinations of metal ions and organic linkers to tailor selectivity towards the respective pollutants. By selective absorption, several mechanisms can be active between MOFs and pollutants, such as electrostatic interaction, hydrogen bonding, or coordination [44,53]. Thirdly, MOFs can function as catalysts in advanced oxidation processes by generating reactive species, enabling the degradation of organic pollutants. However, since pure MOFs are typically obtained as fine powders with lower mechanical stability, they are often incorporated into composite materials in different macroscale forms, such as pellets, beads, and membranes, which facilitates their integration into water treatment systems [54,55]. Despite the apparent advantages of using MOFs in water remediation, their direct application remains challenging, as many conventional MOFs lack the stability to withstand prolonged exposure to aqueous environments due to weak coordination within the metal-ligand framework [56,57,58]. Another significant challenge in MOF applications is their recovery and recyclability during water purification. Many MOFs are synthesized in the form of micro/nano-crystals or fine powders, making it difficult to separate them from the solution after successful pollutant adsorption due to their small size and their sensitive nature to harsh desorbing agents or processes [53,59]. Since regeneration of MOFs requires specific conditions, such as high temperature, their framework could be damaged and, in consequence, the adsorption capacity could be reduced in subsequent cycles [60]. These issues, taken together, limit the commercial viability of MOFs under realistic water treatment conditions.

Recent studies have shown that the design of water-stable MOFs by employing high-valent metal ions, such as Zr^4+^, Ti^4+^, Al^3+^, or Cr^3+^, can address the problems of water instability and low recyclability. These metals exhibit strong oxygen-metal coordination with carboxylate linkers or other coordination ligands, improving their stability in water. Another design approach relies on the incorporation of more hydrophobic ligands into the framework, which can reduce water accessibility and lead to an eventual weakening of interactions within the MOF framework [41,57].

### 1.4. The Synergy of Lignocellulosic Biomass and MOFs

MOFs and LCB represent two versatile platforms, and their combination into composite materials offers a compelling strategy for creating sustainable, high-performance composites. LCB-MOF composites combine the structural advantages of MOFs, like their high porosity, tunable chemistry, and large surface area, with the renewability, low cost, and functional richness of lignocellulose. The practical applicability of MOFs is significantly enhanced by forming composites with LCB, as LCB can act as a scaffold, immobilizing MOF particles within a macroscopic, mechanically robust structure that is easily regenerable and recyclable. This hybrid composite improves hydrothermal and mechanical stability, suppresses particle loss and metal leaching, which significantly facilitates realistic water remediation [61,62]. As a result, LCB-MOF composites bridge the gap between the exceptional intrinsic properties of MOFs and the practical application of scalable, sustainable wastewater systems. An emerging sustainable strategy in LCB-MOF composite design involves using LCB fractions or their derivatives not only as substrates but also as direct precursors for MOF synthesis. This method is often referred to as a green approach, in which biomass-derived molecules can act as coordinating ligands during MOF formation, strictly reducing the use of petrochemical reagents [63]. Literature reports on lignin-derived organic ligands exist, but direct use of cellulose hydrolysate or hemicellulose as MOF ligands remains a poorly explored research area in LCB-MOFs.

Cellulose, particularly in the form of cellulose nanocrystals, can provide a highly effective scaffold for MOF growth. Hydroxyl groups on cellulose chains can act as nucleation sites for metal ions, enabling *in situ* MOF formation and preventing agglomeration of MOF particles. Moreover, when MOFs are incorporated into a cellulose matrix, the resulting composites become easily adaptable to macro-structures due to their structural integrity, which further facilitates their recovery and reuse [62,64].

Besides cellulose, hemicelluloses consist of branched polysaccharides rich in hydroxyl and carboxyl functional groups, which could coordinate with metal ions and serve as a flexible scaffold for MOF nucleation and deposition via coordination interactions and hydrogen bonding, respectively. However, its amorphous nature, inferior mechanical properties, and poor stability compared to cellulose-based systems present obstacles for its usage in MOF synthesis [65,66]. Hemicelluloses were successfully utilized in water remediation in the form of hydrogels, but their low thermal and mechanical stability, as well as high swelling ability, present obstacles for successful applications [67]. To the best of the authors’ knowledge, there are no published works in which hemicelluloses have been involved in the synthesis of LCB-MOFs.

Lignin exhibits strong π–π interactions and contains hydrophobic domains that stabilize MOF particles in aqueous environments. Moreover, the combination of lignin and MOFs demonstrated to affect water stability with reduced leaching of metal ions, while the aromatic skeletal structure of lignin contributes to enhanced selectivity for organic dyes using π–π stacking interactions [68,69,70]. From a sustainability perspective, LCB-MOF composites offer a green approach that enhances MOFs’ stability and recyclability and reduces the amount of MOFs needed for satisfying adsorption rates, and, therefore, eventually reduces chemical pollution [61].

## 2. Synthesis Strategies of Lignocellulosic Biomass-Based MOFs

**Figure 2:**
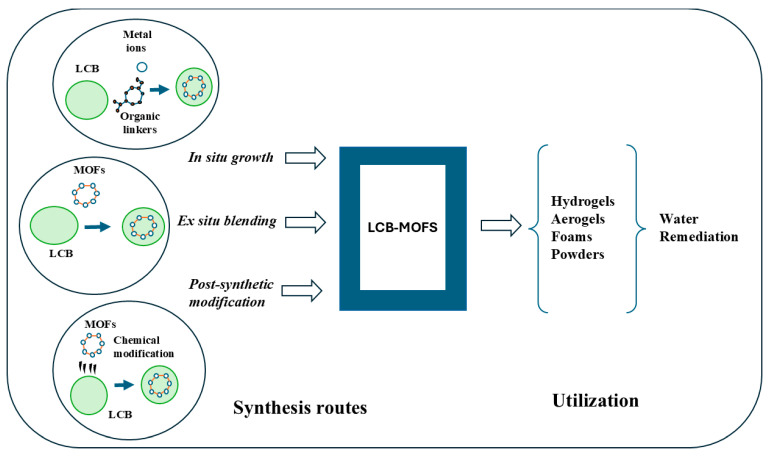
Synthesis strategies of LCB-based MOFs for water remediation.

Depending on the interaction between the biomass matrix and MOFs, various synthetic strategies have been developed, which are classified into *in situ* and ex situ growth methods and post-synthetic modifications (Figure 2). Each manufacturing method offers different stability, bonding, and performance of the synthesized MOF composites, giving respective advantages for wastewater remediation applications.

### 2.1. In Situ Synthesis

**Figure 3:**
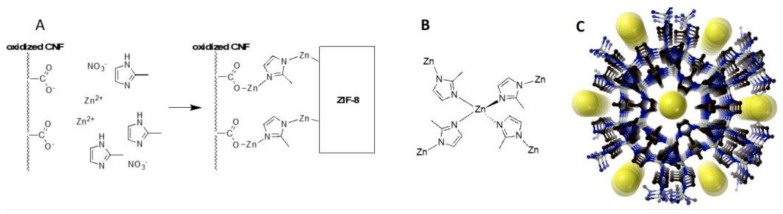
Figure reproduced from Thunberg et al. [73], under the terms of the CC BY license. (**A**) Synthesis of ZIF-8 on oxidized CNF in water. (**B**) The tetrahedral coordination of Zn in ZIF-8. (**C**) Structural representation of ZIF-8 (Cambridge Structural Database code FAWCEN) with the cavities represented by yellow spheres and hydrogen atoms omitted for clarity.

*In situ* growth is one of the most widely used techniques for incorporating MOFs in LCB, especially within cellulose scaffolds [71]. This method involves nucleating and crystallizing MOF particles directly inside the biomass matrix by leveraging surface functional groups, such as carboxyl, hydroxyl, and phenolic groups, as coordinating sites for metal ions (Figure 2). The direct crystallization technique within cellulose matrices leads to strong interfacial bonding and well-dispersed MOF particles, which enhance overall mechanical stability and structural integrity [62,72]. Recent studies investigated MOFs nucleation and early-stage growth on TEMPO-oxidized cellulose nanofibrils (TO-CNFs) at the molecular level. Thus, Zhang et al. reported that carboxylic groups on TO-CNFs form coordination-like bonds with metal ions, and hydroxyl groups of TO-CNFs exhibit hydrogen bonds with the MOF linkers. These dual interactions provided nucleation points for MOF formation and additionally facilitated the growth of larger MOF clusters on the fiber substrate [72]. Similarly, in the study by Thunberg et al., a hybrid cellulose-MOF material was fabricated by self-assembly of zinc-based zeolite imidazole framework (ZIF) nanocrystals on oxidized cellulose nanofibrils in aqueous medium (Figure 3) [73]. Beyond adsorption applications, *in situ* grown MOFs have also been used in an innovative strategy, producing a hierarchical, porous, and recyclable immobilized enzyme-MOF-wood hybrid system for sustainable water remediation. Chen et al. prepared this system by *in situ* growth of the copper-based MOF HKUST-1 within a modified delignified wood scaffold, followed by metal exchange from copper to iron, giving the MOF HP-MIL-100(Fe), and immobilized horseradish peroxidase for the degradation of phenol in wastewater [74].

### 2.2. Ex Situ Synthesis

Another scalable strategy for fabricating LCB-based MOFs is the ex situ approach. The ex situ method involves impregnating MOF powders into the biomass matrix through a multi-step process. The first step involves conventional MOF synthesis, while in the second step, the MOFs are loaded into the biomass matrix (Figure 2). The third final step consists of processing the LCB-MOF composite into the desired form using common techniques, such as solvent casting, vacuum impregnation, freeze-drying, mechanical stirring, or co-precipitation [75]. The use of ex situ pre-formed MOFs helps with maintaining their structural characteristics, porosity, access to active sites, and controlling MOF loading within the biomass matrix, achieving higher loadings compared to in situ methods, when successively blending with biomass. Therefore, the fabricated materials exhibited a large surface area and high adsorption capacity [75]. For example, Zhang et al. fabricated a fluorescent composite hydrogel loaded with the zeolitic imidazolate framework ZIF-8 by ex situ synthesis, composed of CNFs, where carbon dots as well as ZIF-8 units were grafted on and embedded in a cross-linked acrylamide hydrogel, for the detection and adsorption of tetracycline (TC) [76]. More recent research has shown that Zr-based or zeolite imidazolate frameworks can be physically loaded into cellulose-based gels or membranes and used successfully for pollutant removal. These composites often showed improved adsorption capacities, mechanical stability, and recyclability compared to pure MOF powders alone [77,78]. However, the ex situ approach has several limitations, including the need for multiple synthesis steps, prolonged processing times, and the use of hazardous organic solvents like DMF or DEF in their conventional synthesis protocols, which are generally toxic to the environment [79]. Furthermore, the interaction between biomass and MOFs in physically mixed composites generally involves weaker bonds, such as hydrogen or van der Waals forces, which can lead to MOF leaching during their utilization [80]. Other disadvantages include difficulty achieving homogeneous MOF distribution within the support, leading to aggregation of MOF particles on the surface of the support, which offers high accessibility but results in generally low MOF contents in the prepared composites [81,82].

### 2.3. Post-Synthetic Modification

Post-synthetic modification is a versatile strategy for synthesizing LCB-based MOFs, in which MOFs can be synthesized separately under optimal conditions to form their crystalline and pore-defining structures [83]. After that, these pre-synthesized MOFs can be combined with LCB biopolymers through chemical modification of the functional groups of either partner (Figure 2). Post-synthetic strategies can also introduce new functionalities, such as amines (-NH_2_), thiols (-SH), and carboxylates (-COOH), which can improve adsorption characteristics and selectivity towards water pollutants [44].

LCB materials are inherently rich in functional moieties, such as hydroxyl, carboxyl, phenolic, or methoxy groups, which can strongly interact with MOFs. For example, cellulose nanofibrils or microfibers can be surface modified by oxidation or carboxylation to introduce additional anchoring sites for MOFs. These functional groups can readily engage in electrostatic interactions and form hydrogen bonds with MOF linkers or metal nodes [84]. Therefore, MOFs such as Zr-based UiO-66, which possess oxygen-containing groups (Zr-OH), can be physically crosslinked with the hydroxyl groups of cellulose via hydrogen bonding, which, in fact, helps to minimize UiO-66 leaching in adsorption experiments. Further, the carboxylate groups of cellulose coordinate with Zr(IV) centers in the UiO-66 framework, forming strong interfacial interactions that anchor the MOFs to cellulose and yield a stable composite that combines the mechanical robustness of cellulose with the high porosity of MOFs [85,86].

Post-synthetic modification offers clear advantages from the direct blending of cellulose supports and MOF powders, which often results in heterogeneous distribution and weak adhesion. In particular, the doping process allows the MOFs to be integrated into the cellulose matrix. Two post-assembly strategies are sol–gel or electrospinning of MOFs dispersed in cellulose dispersions, followed by freeze-drying [83]. Both electrospinning [87] and sol–gel processing [88] allow controlled integration of MOF particles into the LCB substrate, enabling uniform dispersion, strong interfacial bonding, and enhanced structural integrity. Since mechanical mixing can lead to loss of MOF particles during the desired application, the affinity and compatibility of cellulose and MOFs can be improved by post-modification of cellulose fibers via carboxymethylation, etherification, or sulfation. Additionally, the grafting of MOFs with amine and carboxylic functional groups can promote interaction between cellulose and MOFs [83].

An important challenge of post-synthetic modification is the restriction of potential pore blockage during synthesis, which can occur when grafting or coating is too dense or poorly controlled, interfering with the internal MOF pore network and reducing its effective surface area [73]. Another characteristic is the chemical stability of the bond between biomass and MOFs. Covalent interactions offer good performance but are synthetically difficult, while non-covalent interactions (hydrogen bonding, π–π stacking) are simpler to implement but can be weakened in harsh environments (pH changes) [64,89].

## 3. LCB-MOF Composites for Wastewater Remediation

This section provides an in-depth exploration of recent advances of LCB-based MOFs in water remediation. It emphasizes the role of cellulose scaffolds for MOF synthesis and that of lignin-derived ligands in the formation of biobased MOFs. Furthermore, the chemical interactions, structural properties, and adsorption characteristics of these MOFs are discussed in the frame of water remediation applications (Table 1).

### 3.1. Cellulose-Based MOFs (Cellu-MOFs)

**Figure 4:**
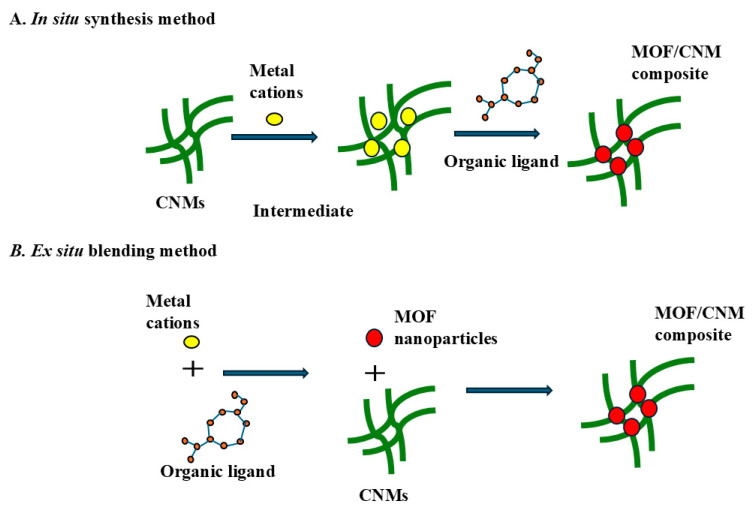
Schematic representation of two methods to prepare cellulose nanomaterial (CNM) MOF composites.

Cellulose is used mainly in its nanoscale form as cellulose nanomaterials (CNMs) as a substrate for fabricating cellulose-based MOF composites. Cellulose derivatives such as nanocellulose possess abundant hydroxyl groups, which coordinate with metals to form biobased MOF composites and offer possibilities for functionalization [101]. Both CNFs and cellulose nanocrystals (CNCs) were employed to produce aerogels as adsorbents. However, CNFs are considered more effective due to their fibrous and flexible nature [102]. In the literature, in situ and ex situ approaches are reported for the synthesis of CNM/MOFs composites (Figure 4). When using CNM as a supporting matrix, particularly in situ synthesis is preferred because of the presence of negatively charged groups on the surface of CNM, which can act as a template to bind metal ions and eventually initiate MOF growth with present organic ligands directly inside the CNM substrate, keeping the surface of the produced composites free from contamination [80,103].

Cellu-MOFs composites, after homogenization, can be processed into various materials, such as beads, gels, and foams, to be used as adsorbents for water remediation. Zr-based MOFs in a cellulose matrix were realized by in situ synthesis of UiO-66 and UiO-66-NH_2_ on a modified cellulose sponge via the coordination of Zr^4+^ metal ions and negatively charged functional groups on the cellulose scaffold. The resulting composites maintained their dimensional stability in water and successfully adsorbed MB dye and mercury ions (Hg^2+^) [98].

Strategies to address the challenges associated with MOF recovery were presented by various studies. Lee et al. fabricated composite beads composed of carbonized and activated ZIF-8 immobilized in cellulose hydrogel beads, resulting in a composite material with a surface and high maximum adsorption capacity for Rhodamine B (RB) dye from water (Table 1) [78]. Ma et al. developed a foam composite material by embedding cellulose fibers, functionalized with in situ grown ZIF-8, in a CNF suspension, followed by freeze-drying. The prepared foams showed good mechanical performance and high adsorption capacity for RB, heavy metal ions, and organic solvents [97]. Meng et al. developed a biodegradable Cellu-MOF aerogel from a pre-synthesized composite of chitosan (CH) and CNFs, which was soaked first in a Zn^2+^ and then in a 2-methylimidazole solution for in situ formation of ZIF-8 inside the aerogel network. The developed composite showed a relatively high surface area and achieved a satisfying absorption capacity for Cu(II) ions [91]. Similarly, Ren et al. prepared a hybrid aerogel as a catalyst for the activation of peroxymonosulfate by incorporating ZIF-9 and ZIF-12 into a cellulose aerogel to degrade organic pollutants such as RB, TC, and *p*-nitrophenol in water. Further, it was shown that the recycled aerogels did not lose adsorption performance over three consecutive cycles [90].

The combination of ZIF-8 and CNF reported by Nabipour et al. produced flame-retardant aerogels, which showed adsorption performance for organic solvents and oils [104]. Bo et al. fabricated a hybrid composite aerogel from ZIF-8 and cellulose in an ex situ method, which showed improved adsorption characteristics compared to cellulose aerogel or ZIF-8 alone [95]. Moreover, Wen et al. fabricated a PTA/ZIF-8@Cellulose aerogel for the photocatalytic degradation of organic dyes such as MB and RB by adding ZIF-8 as a filler in the composite materials. It showed fast, effective, and stable catalytic activity for both MB and RB, even after 5 photocatalytic cycles [96].

**Figure 5:**
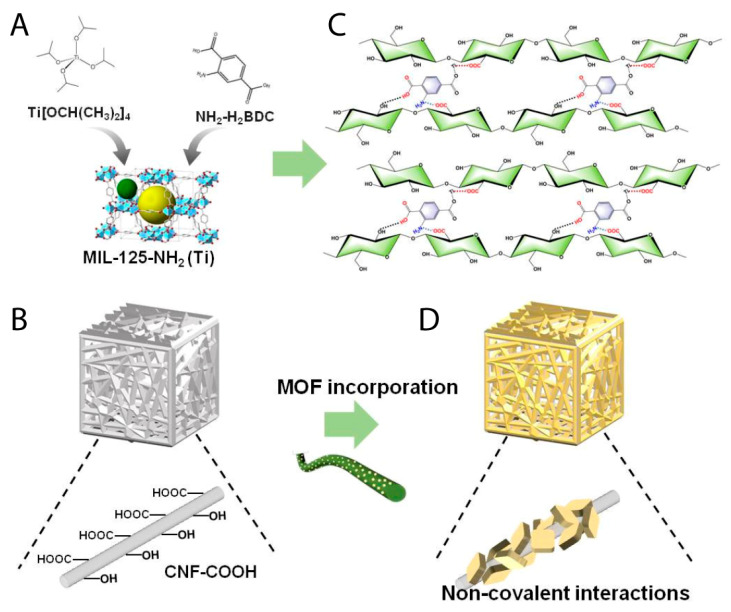
Schematic illustration of the fabrication process of MIL-125-NH_2_/CNFs aerogels. (**A**) MIL-125-NH_2_(Ti), prepared from titanium isopropoxide and 2-aminoterephthalic acid. (**B**) TEMPO-oxidized CNFs. (**C**,**D**) Anchoring and dispersion of Ti-MOFs in TEMPO-oxidized CNFs by non-covalent interaction between the amino groups of the organic ligand and carboxylic groups on the C_6_ position of the glucose units of CNFs. Figure reprinted from Yang et al. [94], licensed under CC BY 4.0.

A recent study has reported an *ex situ* protocol to produce Cellu-MOFs aerogels containing Ti-based MOFs (MIL-125-NH_2_) and carboxylated CNFs, resulting in a stable adsorptive aerogel for the effective photocatalytic removal of Cr(VI) ions (Figure 5) [94]. Cellulose isolated from biomass was widely used as a scaffold for zinc-based MOF (MOF-5) growth, enabling dispersion and mechanical stability in a composite such as MOF-5/cellulose aerogels for dye adsorption [93].

### 3.2. Lignin-Based MOFs (Lig-MOFs)

Lignin has potential as a ligand for MOF synthesis due to its variety of functional groups that can coordinate with metal ions, but its direct use as an organic ligand is limited by its high molar mass and inhomogeneous, amorphous structure. One prominent route is to derive organic linkers from lignin. Lignin can be depolymerized into smaller fragments with phenolic or carboxylic hydroxyl moieties using deep eutectic solvents that can easily coordinate with metal ions (e. g., Zr^4+^, Fe^3+^) to form MOFs or MOF-like structures. These lignin-derived MOFs contain aromatic backbones, which provide unique functionality, such as enhanced π–π interactions, thermal stability, and hydrophobicity [105]. Further, lignin adds antioxidant and antimicrobial properties to the prepared MOFs, which can enhance their water remediation potential [106].

Thus, the literature reports on MOFs where lignin is used directly as a ligand, to the best of the authors’ knowledge, are not yet available. However, lignin-derived compounds, such as ferulic acid (FA), vanillin, and caffeic acid, have been tested as ligands for the preparation of biobased MOFs [24]. FA was used to form MOFs with different metal nodes, such as Zn(I) [107], Zn(II) [108], Cu(II) [109], and vanillin was similarly utilized as a ligand to form MOFs with Zn(II) [110] and Cu(II) [111], but specific applications related to the adsorption of potential water contaminants are missing from their respective studies. Among these reports, only Zhou et al. designed an FA-based MOF for wastewater remediation, synthesizing a Zn-MOF containing FA for the photodegradation of organic dyes. In this synthesis, a strong bond formed between asymmetrical Zn^2+^ ions and deprotonated phenolic and carboxylic hydroxyl groups of FA. The study showed the successful photocatalytic degradation of methyl violet (MV) and RB in aqueous solution under UV radiation [108].

**Figure 6:**
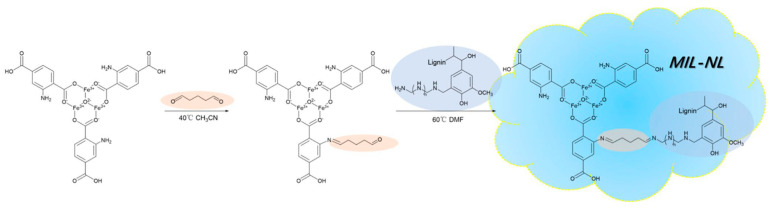
Route to the synthesis of MIL-NL. Adapted with permission from Wang et al. ACS [100]. Copyright (2026) American Chemical Society.

Following the strategy of using lignin as a support material, Wang et al. fabricated lignin-based MOFs by a grafting approach, introducing aminated lignin into a zirconium cluster-based MOF (UIO-66-NH_2_) using glutaraldehyde as a cross-linker. The resulting MOF UIO-g-NL maintained its original crystal structure and functioned effectively as a selective adsorbent for methyl orange (MO) from a mixed solution containing both MO and MB. The addition of aminated lignin enhanced π–π stacking, NH-π interactions, and electrostatic attraction between UIO-g-NL and MO [99]. Furthermore, they elaborated their idea by grafting aminated lignin onto MIL-101-Fe-NH_2_, which contains not only the crystal structure of MIL-101-Fe-NH_2_ but also that of Fe_2_O_3_. The presence of amine and hydroxyl groups of the aminated lignin on MIL-NL benefits in the hydrogen bonding with different chemical species. Altogether, MIL-NL showed improved electrostatic and π–π interactions and satisfying adsorption capacities for CR from water (Figure 6) [100]. Moreover, Wang et al. synthesized a phenolated lignin-containing Zr-based MOF via a one-pot approach and reported effective removal of MB and MO with satisfactory reusability, maintaining adsorption efficiencies of >97% after six cycles [70].

## 4. Key Characteristics and Performance Enhancement

The incorporation of MOFs into the LCB matrix resulted in hybrid composites with improved structural integrity, functional performance, and stability with respect to pure MOFs. This section examines the key morphological, structural, and stability-related characteristics of LCB-MOF composites, encompassing how LCB mitigates common MOF limitations.

### 4.1. Morphological and Structural Properties

**Figure 7:**
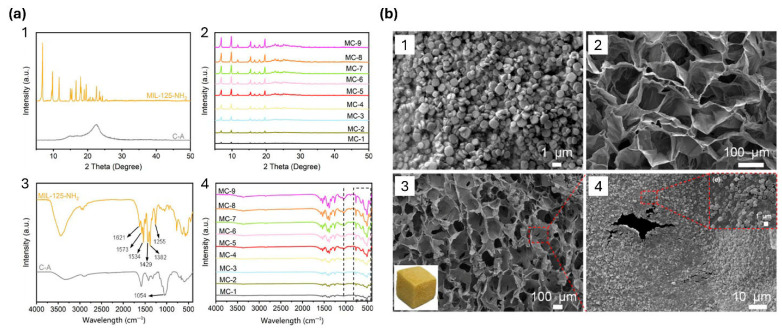
Figure reproduced from Yang et al. [94], licensed under CC BY 4.0. Summary of results of the characterization of the porous MIL-125-NH_2_ and nanocellulose composite aerogel. (**a**) The obtained XRD patterns (1, 2) and FTIR spectra (3, 4) of the CNF aerogel (cellulose aerogel (C-A), MIL-125-NH_2_, and composite aerogels (MC-1, …, MC-9). (**b**) SEM images of (1) pristine MIL-125-NH_2_ powder, (2) C-A aerogel, (3, 4) hybrid MC-5 aerogel, with high-resolution view of MC-5 (insert).

The successful formation of LCB-MOF composites is typically confirmed with a combination of microscopic and spectroscopic analysis, including transmission electron microscopy (TEM), scanning electron microscopy (SEM), Fourier-transform infrared spectroscopy (FTIR), X-ray diffraction (XRD), and Brunauer–Emmett–Teller (BET) surface area analysis (Figure 7). SEM and TEM images are commonly used to visualize MOF crystal distribution on biomass surfaces and to confirm uniform dispersion on cellulose fibers, lignin nanoparticles, or bioderived aerogels [73,91,112]. Compared to aggregated MOF powders, LCB-MOFs showed reduced particle agglomeration and more homogeneous dispersion, as the MOF grows inside the scaffold [113]. XRD analysis verifies that the crystalline structure of MOFs is retained after composite formation. In most reported works, characteristic diffraction peaks of MOFs such as ZIF-8, UIO-66, and MIL-101, respectively, remain unchanged, indicating that physical mixing, impregnation, or in situ growth on LCB scaffolds do not disturb MOF crystallinity [97,112]. In Lig-MOFs, a new peak compared to pure MOFs was observed in the respective XRD patterns, indicating, as suggested by the authors, that lignin had an influence on the formation of the MOF crystals via accelerating deprotonation of the ligand and promoting MOF particle nucleation [114].

Additionally, FTIR spectroscopy showed that the interfacial interactions between MOFs and LCBs result in characteristic shifts in functional group IR bands. In the work of Lu et al., cellulose-supported magnetic Fe_3_O_4_-MOF composites for enhanced dye removal exhibit characteristic vibrational band shifts, such as for ester bonds, indicating that the used organic ligand benzenetricarboxylic acid was anchored on the cellulose substrate via ester bonds and eventually coordinated with the metal nodes for MOF formation [115]. The same observation was made by Yang et al., who prepared cellulose paper containing calcium carbonate filler and modified with in situ grown MOF-5, the added calcium carbonate reduced hydrogen bonding between cellulose fibers, thus exposing more OH groups for reaction with the carboxylic acid groups of the organic ligand benzenedicarboxylic acid, resulting in smaller MOF crystals compared to when non-modified cellulose fibers were used [116]. For Lig-MOFs, FTIR spectra provide indications for lignin-metal interaction by shifted IR bands of lignin carboxylic OH groups, due to coordination with Zr(IV) ions of the utilized MOF [70].

From a structural perspective, LCB plays a vital role as a scaffold by preventing the aggregation of MOF particles during synthesis and processing steps. CNFs and lignin-rich matrices form an interconnected porous network, which physically divides MOF particles and promotes hierarchical porosity of MOF micropores and LCB macropores 100,101. BET analysis carried out in the study of CNF/ZIF-8 and pure ZIF-8 by Thunberg et al. showed that the total surface area of the composite was slightly lower than for pure ZIF-8, but the accessible pore volume and mass-transfer efficiency were improved by the incorporation of biomass, resulting in a composite with reduced pore blockage and enhanced microporosity [73].

### 4.2. Stability and Reusability

The primary challenge to the large-scale application of MOFs in wastewater remediation is their poor stability in aqueous environments. According to literature, many MOFs suffer from framework collapse and metal ion leaching after prolonged exposure to water, acidic/basic conditions, or repeated regeneration cycles [117]. The incorporation of LCB has shown significant improvements in preventing these issues through multiple mechanisms. Firstly, the physical confinement of MOF crystals within the LCB matrix reduces direct exposure to water, limiting hydrolysis of metal-ligand bonds [112]. Secondly, strong interfacial interactions, such as hydrogen bonding, electrostatic interactions, or coordination between OH groups of LCB components and the metallic centers of MOFs, can reduce structural degradation [105]. For example, Zr-based MOFs (UIO-66) immobilized on cellulose showed greater hydrothermal stability compared to unsupported MOFs [85]. Furthermore, when lignin acts as a partner in MOF composites, its abundant phenolic and carboxyl functional groups can coordinate with metal centers, which helps to stabilize the coordination framework and reduce hydrolysis in aqueous environments. Also, lignin’s polymeric nature provides mechanical performance to the metal framework [70,105]. Recent studies reported that lignin-based MOFs retained a large proportion of their original adsorption capacity after prolonged exposure to water, acidic, or low-alkaline conditions compared to conventional MOFs in similar conditions [61,118].

Another critical parameter is the regeneration and reusability of MOFs. Several studies reported that LCB-MOF composites retain high adsorption capacity over multiple adsorption–desorption cycles. For example, cellulose-based MOF aerogels, beads, and membranes have shown stable adsorption performance over 5–10 cycles for the removal of heavy metal ions, pharmaceuticals, and organic dyes, due to improved mechanical performance and ease of regeneration from aqueous solutions [90,112,119].

## 5. Challenges and Future Perspectives

### 5.1. Current Challenges

One of the major challenges related to LCB-MOF composites is their scalability. As reported, synthesis routes for LCB-MOF composites, such as in situ growth, multi-step functionalization, or freeze-drying, were time-consuming, expensive, and required intensive effort and energy. Additionally, the use of organic ligands, metal salts, and organic solvents can increase material costs, partly reducing the sustainability-related benefits offered by biomass substrates [105,117]. For commercialization, the development of a low-cost, continuous, and green synthesis route is essential.

Most of the adsorption studies were carried out in synthetic wastewater containing a single or a certain type of contaminant. In contrast, real wastewater contains complex matrices with competing ions, pH fluctuations, and natural organic matter, and pollutant concentrations vary. These factors can effectively reduce adsorption efficiency, block active pore sites, and destabilize MOF structures [117]. Therefore, the adsorption activity of LCB-MOF composites under realistic environmental conditions remains insufficiently discussed.

Although LCB-MOFs were synthesized from renewable materials, their biocompatibility needs to be tested for future applications. The available bio-safety evaluations, such as in vitro studies, suggest that several biobased MOFs exhibited low cytotoxicity and good biocompatibility; however, they require tests, such as long-term in vivo studies, to provide comprehensive evidence for real-world applications [105]. Furthermore, synthesis protocols for biobased MOFs that avoid the introduction of toxic metal ions, such as cobalt (Co) [120] and cadmium (Cd) [121], tend to yield promising materials. Thus, combining lignin derivatives with metal cations such as Zn^2+^, Fe^2+^, Ca^2+^, and Cu^2+^ gives biobased MOFs with good biosafety and biocompatibility [105]. Finally, the use of residual organic solvents, such as methanol or DMF, during the synthesis of LCB-MOFs may lead to safety issues [79].

Moreover, the incorporation of LCB can enhance hydrothermal stability, another critical parameter for the practical application of MOFs, which describes the ability to withstand prolonged exposure to an aqueous environment without structural degradation [122]. However, long-term durability remains a challenge in harsh wastewater conditions. Prolonged exposure to water, repeated adsorption–desorption cycles, and mechanical stress may lead to MOFs degradation, metal ion leaching, or LCB structure collapsing [123]. Thus, it is essential to improve long-term stability without compromising the porosity and activity of LCB-MOFs.

### 5.2. Future Research Directions

Future research activity should focus on underutilized or non-traditional LCB resources, such as agricultural waste, forestry waste, algae-derived polysaccharides, and industrial by-products. These materials could provide unique structural properties or active functional groups that can enhance MOF characteristics and selective pollutant degradation [124]. For example, in a recent study, residual LCB in the form of African Star Apple seed pods was used as feedstock to prepare a MOF-based composite for water remediation. The biochar-MOF nanocomposite prepared via ball milling exhibited high adsorption efficiency for heavy metals and steroid hormones from both synthetic and real wastewater over multiple cycles [125]. Similarly, a novel cellulose@Fe_3_O_4_@ZIF-8 magnetic aerogel adsorbent derived from the fruits of the Syrian mesquite shrub (*Prosopis farcta*) was synthesized for the efficient removal of gasoil from aqueous solution [126].

Additionally, the development of advanced modification strategies represents an effective route to enhance the selectivity of LCB-MOF composite for wastewater renovation. Heteroatom doping in LCB composites improves surface chemistry, improves interactions with specific contaminants, and enhances catalytic activity for advanced oxidation processes [127,128]. Furthermore, optimizing material composite performance can be carried out by computational simulations and structural property relations through rational material design [129].

Combining LCB-MOF composites with other wastewater treatment strategies, such as membrane filtration, electrochemical treatment, and advanced oxidation processes, can lead to a synergistic effect in removing pollutants and improving operational stability. These hybrid systems represent a promising direction for a next-generation water renovation platform [130].

Moreover, comprehensive life-cycle assessments (LCA) and techno-economic analyses (TEA) are needed to address the practical feasibility and environmental impact of LCB-MOF composites. LCA can be used to quantify environmental indicators, such as carbon footprint, energy demand, and resource consumption across the material life cycle, while TEA can identify cost drivers and structural constraints [131,132]. Together, these tools enable information regarding the sustainable use of materials and processes for LCB-MOFs in large-scale implementation.

## 6. Conclusions

LCB-MOF composites are a promising class of sustainable materials for wastewater remediation. By integrating the surface functionality, renewability, and abundance of LCB with the tunable porosity and chemical functionality of MOFs, these innovative materials address key limitations associated with conventional adsorbents. Various synthesis strategies, including in situ growth, composite blending, and ex situ impregnation, have been developed to tailor material structure and performance. LCB-MOF composites demonstrate enhanced adsorption efficiency, improved aqueous stability, and superior recyclability compared to pure MOFs, enhancing their potential for practical environmental applications. Despite their promises, challenges remain for LCB-MOFs in scalability, wastewater remediation performance under real-world conditions, and long-term stability. Future research must focus on rational material design and system integration to enhance their performance in industry. Overall, LCB-MOF compounds offer versatile pathways within the framework of circular economy principles and green chemistry solutions, enabling the valorization of biomass waste into high-value-added functional materials for sustainable water remediation.

## Figures and Tables

**Table 1:** Different forms of LCB-MOFs for wastewater remediation (q_e_ = adsorption capacity at equilibrium, q_max_ = maximum adsorption capacity).

Form	Composition	Preparation Method	General Properties	Adsorption Performance	Pollutant	Ref.
Aerogel	ZIF-9, ZIF-12, cellulose	In situ	Avg. pore size: 45.353 nm (ZIF-9/cellulose aerogel) 75.798 nm (ZIF-12/cellulose aerogel)	Removal: 99% Rhodamine B (RB), 90% tetracycline (TC) 90% p-nitrophenol	RB, TC, p-nitrophenol	[90]
Aerogel	ZIF-8, chitosan (CS), cellulose nanofibers (CNFs)	In situ	Specific surface area: 206 m^2^/g Porosity: 91%	q_max_ (Langmuir): 245 mg/g	Cu(II)	[91]
Aerogel	UiO-66, nanocellulose	Ex situ	Specific surface area: 826 m^2^/g	q_e_: 71.7 mg/g (Methyl Orange (MO)) 51.8 mg/g (Methylene blue (MB))	MO, MB	[92]
Aerogel	MOF-5, cellulose	Ex situ,	Pore size: 2–50 nm	q_max_ (Langmuir): 60 mg/g	Acid Blue	[93]
Aerogel	MIL-125-NH_2_, CNFs	Ex situ	Specific surface area: 582 m^2^/g Pore volume: 0.38 cm^3^/g	Removal: 99.8%	Cr(VI)	[94]
Aerogel	ZIF-8, cellulose	Ex situ	Pore volume: 19.33 cm^3^/g Porosity: 95.3%	q_max_ (Langmuir): 41.8 mg/g Removal: 90–99.9% (10–1 mg/L)	Cr(IV)	[95]
Aerogel	ZIF-8, cellulose, phosphotungstic acid (PTA)	Ex situ	N/A	Removal: 99.8% (MB, 10 mg/L, 15 min) 99.7% (RB, 10 mg/L, 60 min)	MB, RB	[96]
Hydrogel	ZIF-8, CNFs, carbon dots	Ex situ	Specific surface area: 14.86 m^2^/g	q_max_ (Langmuir): 810.36 mg/g	TC	[76]
Beads	ZIF-8, regenerated cellulose	Ex situ	Specific surface area: 1412.8 m^2^/g	q_max_ (Langmuir): 565.13 mg/g Removal: 99%	RB	[78]
Paper	ZIF-8, cellulose paper	Ex situ/In situ	Specific surface area: 6–230 m^2^/g Pore volume: 0.01–0.14 cm^3^/g	q_e_: 66.2–354.0 mg/g	Cd(II), Cu(II), Fe(III), Pb(II), Co(II)	[77]
Paper	UiO-66-NH_2_, cellulose fibers	In situ	N/A	Removal: 78.2% Cr(VI) 84.5% MO	Cr(VI), MO	[85]
Foam	ZIF-8, cellulose, CNFs	In situ	Specific surface area: 475.5 m^2^/g	q_e_: 24.6 mg/g (RB) 35.6 mg/g (Cr (VI)) 45.2 mg/g (dimethylformamide (DMF))	RB, Cr(VI), DMF	[97]
Sponge	UiO-66, UiO-66-NH_2_, CNFs	In situ	Specific surface area: 46.6 m^2^/g (UiO-66) 66.4 m^2^/g (UiO-66-NH_2_S) Porosity: 98.8% (UiO-66) 98.7% (UiO-66-NH_2_)	q_e_: UiO-66: 195.1 mg/g (Hg(II)) 294.7 mg/g (MB) UiO-66-NH_2;_ 224.5 mg/g (Hg(II)) 400.9 mg/g (MB)	Hg(II), MB	[98]
Powder	UiO-66-NH_2_, aminated lignin	Post-synthetic modification	Avg. pore size: 1.649 nm Micropore volume: 0.19 cm^3^/g Specific surface area: 511.05 cm^2^/g	q_e_: 1126.7 mg/g (MB) 961.5 mg/g (MO)	MO, MB	[99]
Powder	MIL-101-Fe-NH_2_, aminated lignin	Post-synthetic modification	Specific surface area: 12.4 m^2^/g	q_e_: 1495.23 mg/g (CR); q_max_ (Langmuir): 1345.07 mg/g	CR	[100]
Powder	UiO-66-NH_2_, phenolated lignin	Post-synthetic modification	Pore size: 1.663 nm Specific surface area: 1192.1 m^2^/g	q_e_: 207.04 mg/g (MO) 243.31 mg/g (MB)	MO, MB	[70]

## Data Availability

No new data were created or analyzed in this study. Data sharing is not applicable to this article.
